# Supplementary material for: Backbone 3.0: An R package for extracting network backbones
Source: PLoS One. 2026 May 15;21(5):e0349258. doi: 10.1371/journal.pone.0349258 (PMC13178991; doi:10.1371/journal.pone.0349258)
Supplement: Attachment — Submitted filename: Response to Reviewers.pdf [file pone.0349258.s003.pdf]

Summary of Revisions to "backbone 3.0: An R package for extracting network backbones" (PONE-D-26-02567)

Dear Dr. Wei,

Thank you for obtaining these very helpful reviewer comments, and for the opportunity to submit a revised version of this manuscript. In this cover letter I briefly summarize the revisions I made to address the reviewers' feedback.

1 Editor

1.1 "Please ensure that your manuscript meets PLOS ONE's style requirements, including those for file naming."

I have reviewed the formatting requirements for PLOS One, and have ensured that the manuscript, figures, and file names are in compliance.

1.2 "Please note that PLOS One has specific guidelines on code sharing for submissions in which author-generated code underpins the findings in the manuscript. In these cases, we expect all author-generated code to be made available without restrictions upon publication of the work."

I confirm that all code is available without restriction. It is located in the repository at `https://osf.io/rx6af` and was also uploaded in the manuscript submission system. In the previous round, both reviewers confirmed that I made all data underlying the findings in their manuscript fully available.

1.3 "We are unable to open your Supporting Information file [backbone3.R]. Please kindly revise as necessary and re-upload."

I apologize for any dfficulty with the `backbone3.R` file. I have re-uploaded the file with this revised submission. Additionally, this file is also available in the associated repository at `https://osf.io/rx6af`. Please note that if you do not have R or RStudio installed, you may also open and read this file using a text editing software.

1.4 "Please include captions for your Supporting Information files at the end of your manuscript, and update any in-text citations to match accordingly."

The manuscript now includes a caption for the supporting information (specifically, the file `backbone.R`).

2 Reviewer #1

2.1 "The paper is technically sound and demonstrates the package's functionality through well-designed examples."

I am very pleased to hear that you found the examples effective for demonstrating and illustrating the software.

2.2 "While the paper provides detailed technical descriptions of the implemented models, it does not adequately discuss the relative strengths and weaknesses of different backbone extraction models when applied to the empirical network of bill sponsorship from the 108th U.S. Senate."

As I explain in 3.2, when discussing each class of backbone model, I now provide explicit guidance in model selection.

In the empirical example, I use different backbone models to "*illustrate how different backbone extraction methods might be used under conditions where different amounts of data are available*." This goal is now clarified in the extended caption to Figure 5: "*If the source data represent a bipartite network (A1), then a backbone of the weighted projection can be extracted using a model such as the stochastic degree sequence model (SDSM, A2). If the source data represent a weighted network (B1), then a backbone can be extracted using a model such as the disparity filter (B2). If the source data represent an unweighted network, then a backbone can be extracted using a model such as local sparsification (L-Spar, C2).*"

I now also discuss the strengths and weaknesses of the models illustrated in the empirical case in the new section titled "Comparing models and empirical results".

2.3 "When applying these methods to the empirical case, a summary table is suggested to explicitly indicate: (a) whether each model is statistical or structural, (b) computational complexity, (c) its strengths in capturing what kind of patterns or structures, and (d) key limitations."

Thanks for this very helpful suggestion. In the empirical example, I now include a subsection and associated table (Table 3) titled "Comparing models and empirical results" in which I provide and discuss these details.

2.4 "The network figure is not that self-explained and the patterns are a bit messy."

Thank you for this reminder. I have added extended captions for each of the figures to clarify their interpretations.

3 Reviewer #2

3.1 "The paper clearly demonstrates the functionality of the package through illustrative examples and an empirical case study based on U.S. Senate data. Overall, the work has practical value, particularly in providing a unified and actively maintained tool within the R ecosystem."

I am very happy to hear that you found value (especially practical value!) in this manuscript and the associated software.

3.2 "The manuscript would benefit from a more thorough discussion of model selection. It remains unclear when a particular model should be preferred. Including simulation experiments (e.g., varying network density or noise levels) and quantitative comparisons (such as structure recovery or stability) would significantly strengthen the paper and provide useful guidance to users."

At the end of each section discussing a class of backbone models, I now discuss the considerations that are relevant for model selection (with citations to relevant work) and provide user guidance:

- **For weighted networks:** "*In general, all three models implemented in* `backbone_from_weighted()` *–* `disparity`, `lans`, *and* `mlf` *– yield similar backbones and have similar runtimes. Therefore, while any of these models may be appropriate, because it is the most highly-cited and widely-known, the disparity filter is often the better choice and is the default. By implementing multiple models for extracting the backbone from weighted networks,* `backbone` *facilitates further research on their similarities and differences Yassin et al. (2023).*"

- **For weighted projections:** "*The tradeoff of efficiency for power is the primary consideration in choosing between SDSM and FDSM as a model for extracting the backbone from a weighted projection. Given FDSM's potentially long runtime, SDSM is often the better choice and is the default. The other models implemented in* `backbone_from_projection()` *–* `fixedrow`, `fixedcol`, *and* `fixedfill` *– are known to perform worse and generally should not be used. They are implemented in* `backbone` *for their methodological, rather than practical, applications Neal et al. (2021).*"

- **For unweighted networks:** "*When the goal is to preserve or uncover hidden clusters or communities,* `lspar` *is often the better option and is the default, but* `gspar`, `simmelian`, `quadrilateral`, `jaccard`, `meetmin`, `geometric`, *and* `hyper` *often yield similar results. When the goal is to preserve or uncover hidden hubs or high-degree nodes,* `degree` *is often the better option and is the default. Using* `skeleton` *is not recommended because it yields non-reproducible random backbones; it is implemented for its methodological, rather than practical, application. By implementing multiple models for extracting the backbone from unweighted networks,* `backbone` *facilitates further research on their similarities and differences Hamann et al. (2016).*"

3.3 "The role of key parameters (e.g., alpha) is not sufficiently discussed. The sensitivity of results to these parameters is unclear, which may make it difficult for practitioners—especially beginners—to apply the methods appropriately. Providing some guidance or empirical analysis on parameter selection would improve usability."

This is a great suggestion. I now offer the following guidance: "*For statistical models, the value of* `alpha` *should be chosen based on disciplinary conventions and the researcher's tolerance for type-I error, where for most disciplines* `alpha = 0.05` *(the default) or* `alpha = 0.01` *are typical. For structural models, the appropriate value of* `parameter` *is* `model`*- and data-dependent, and should be chosen based on the level of sparsification the researcher desires.*"

3.4 "The computational efficiency of different models is not clearly evaluated. Since some methods may differ substantially in runtime, it would be helpful to include basic performance comparisons to inform practical use."

Thanks for this very helpful suggestion! In general, all backbone models run very quickly. The only exception is the Fixed Degree Sequence Model (FDSM), which is slow because it requires monte carlo simulation. I have revised the manuscript in three ways to clarify this issue:

- When illustrating the SDSM and FDSM, I now explicitly report and compare their runtimes: "*The null model used by the SDSM is computationally efficient but statistically less powerful; it takes only 0.039 seconds to run, but detects fewer statistically significant edges. Conversely, the null model used by the FDSM is computationally intensive but statistically more powerful; it takes 13.131 seconds to run (over 300 times longer), but detects more statistically significant edges.*"

- In the empirical case study, I now explicitly report and compare the runtimes of each of the three illustrated models: SDSM runs in 0.336 seconds, disparity filter in 0.01 seconds, and local sparsification in 0.007 seconds.

- Because I now report practical runtimes, I now also clarify that "*When software run times are reported, they reflect code execution using a MacBook Pro with an Apple M1 Max processor and 64GB of memory.*"

References

Hamann, M., Lindner, G., Meyerhenke, H., Staudt, C. L., and Wagner, D. (2016). Structure-preserving sparsification methods for social networks. *Social Network Analysis and Mining*, 6(1):22.

Neal, Z. P., Domagalski, R., and Sagan, B. (2021). Comparing alternatives to the fixed degree sequence model for extracting the backbone of bipartite projections. *Scientific Reports*, 11(1):23929.

Yassin, A., Haidar, A., Cherifi, H., Seba, H., and Togni, O. (2023). An evaluation tool for backbone extraction techniques in weighted complex networks. *Scientific Reports*, 13(1):17000.
